# Correlation Between Chinese Surgical Nurses’ Perception of Holistic Humanistic Caring and Their Experience of Workplace Violence: A Cross‐Sectional Study

**DOI:** 10.1155/jonm/5884883

**Published:** 2026-08-09

**Authors:** Hongwei Chang, Danli Xiong, Min Tian, Ting Chen, Yuqin Chen, Yao Cheng, Yan Meng, Huantian Li, Yilan Liu, Sai Yang

**Affiliations:** ^1^ Gastrointestinal Surgery, Union Hospital, Tongji Medical College, Huazhong University of Science and Technology, Wuhan 430022, China, hust.edu.cn; ^2^ Hepatobiliary Surgery, Union Hospital, Tongji Medical College, Huazhong University of Science and Technology, Wuhan 430030, China, hust.edu.cn; ^3^ Thyroid and Breast Surgery, Union Hospital, Tongji Medical College, Huazhong University of Science and Technology, Wuhan 430030, China, hust.edu.cn; ^4^ Internal Medicine Department, Sun Yat-sen University Cancer Center, Guangzhou 510050, China, sysucc.org.cn; ^5^ Department of Nursing, Affiliated Hospital of Hubei University of Traditional Chinese Medicine, Wuhan 430060, China; ^6^ Department of Nursing, Jingzhou First People’s Hospital, Jingzhou 434000, China; ^7^ General Surgery, Xiangyang No.1 People Hospital Affiliated to Hubei University of Medicine, Xiangyang 441099, China; ^8^ Department of Nursing, Union Hospital, Tongji Medical College, Huazhong University of Science and Technology, Wuhan 430022, China, hust.edu.cn

**Keywords:** correlation analysis, humanistic caring, workplace violence

## Abstract

**Aims:**

To understand the current perceptions of holistic humanistic caring among Chinese surgical nurses and the prevalence of workplace violence they experience, and to conduct a correlation analysis between them.

**Background:**

The incidence of workplace violence against nurses is high in China, yet preventive measures tailored to nurses’ perspectives remain inadequate. Humanistic caring, as a beneficial resource, requires continuous replenishment to be effectively delivered. Nurses must continually enrich their holistic humanistic caring perception system to sustain compassionate patient caring. Providing compassionate caring to patients and colleagues may help reduce nurses’ risk of workplace violence. However, empirical evidence directly examining the association between nurses’ holistic humanistic caring perception and their experiences of workplace violence remains limited.

**Method:**

In December 2024, a convenience sampling method was employed to select surgical nurses from 30 tertiary and secondary general hospitals across 12 regions in Hubei Province as research participants. The data collection instruments comprised a self‐developed general information questionnaire, the Holistic Humanistic Caring Perception Scale for Nurses, and the Revised Hospital Workplace Violence Survey Questionnaire, each of which demonstrated robust reliability and validity within the context of this study. Data collection was facilitated through the Chinese online platform QuestionStar. Out of 937 distributed questionnaires, 895 valid responses were obtained. Descriptive and inferential statistical analyses were performed using SPSS Version 26.0.

**Results:**

The median (IQR) total score of the Holistic Humanistic Caring Perception Scale among the 895 surgical nurses was 168.00 (151.00, 194.00). The score rates for the six dimensions—self‐caring, hospital managers’ caring, department coworkers’ caring, patients’ caring, family caring, and friends’ caring—were 82.46%, 79.20%, 84.83%, 80.27%, 86.43%, and 84.56%, respectively. In terms of workplace violence experienced by nurses, verbal abuse was the most frequently reported form of WPV, followed by verbal threats, physical assault, and sexual harassment. The findings suggested a negative association between the frequency of various WPV experiences and overall holistic humanistic caring perception among the surveyed surgical nurses.

**Conclusion:**

In this study, 895 surgical nurses surveyed scored above 80% on five dimensions of perceived holistic humanistic caring, with one dimension scoring below 80%. Among these, participants reported the highest level of perceived caring for families and the lowest for the hospital managers, particularly showing deficiencies in hospital management participation. This suggests hospital managers should further streamline channels for nurse participation in management, genuinely implement “shared governance,” and encourage nurses to contribute suggestions for hospital development. This approach would enhance their job satisfaction and sense of belonging while reducing turnover rates. Simultaneously, nurses’ holistic humanistic caring perceptions showed a significant negative correlation with the occurrence of various types of workplace violence. This not only provides empirical evidence and data support for the proposition that enhancing nurses’ holistic humanistic caring perceptions helps reduce incidence of workplace violence but also further suggests that nurses who have experienced workplace violence should receive more comprehensive humanistic caring to alleviate psychological trauma and promote health recovery.

**Implications for Nursing Management:**

Hospital administrators and nursing managers should fully recognize nurses’ need for caring, understand their overall perception of holistic humanistic caring, and continuously provide humanistic caring to nurses while requiring them to deliver humanistic caring to patients, thereby enriching the comprehensive humanistic caring system. Simultaneously, hospitals should deepen the implementation of “shared governance,” opening channels for nurses to voice their concerns. By listening to their innermost thoughts, hospitals can optimize nursing management systems, enhance job satisfaction and professional resilience among nurses, and provide robust support for delivering high‐quality nursing services and improving patient satisfaction.

## 1. Introduction

Workplace violence constitutes a substantial global occupational health issue for healthcare workers and has important implications for workforce safety and healthcare quality [1]. Exposure to workplace violence is associated with adverse psychological outcomes, including anxiety, depression, fear, sleep disturbance, and emotional distress [1–3]. Workplace violence is also linked to reduced job satisfaction, burnout, turnover intention, and impaired quality of care [1, 3]. Nurses are particularly vulnerable to workplace violence because they maintain frequent direct contact with patients and families and often work in high‐pressure clinical environments [1, 4]. Surgical nurses may face additional risks because they care for patients with postoperative pain, complex clinical needs, medication‐related behavioral changes, and high levels of family anxiety [5, 6]. Existing strategies for preventing workplace violence in healthcare settings commonly include zero‐tolerance policies, staff education, environmental safety measures, reporting systems, risk assessment, and organizational protective strategies [1, 7]. However, workplace violence remains difficult to prevent because it is shaped by multiple interacting factors, including patient expectations, communication difficulties, waiting time, staffing levels, resources, and organizational climate [1]. Therefore, in addition to organizational and policy‐level prevention strategies, empowering nurses to maintain caring‐oriented communication and cope with demanding clinical interactions may be an important complementary approach to reducing WPV risk. Research indicates that the demonstration of humanistic caring towards both patients and colleagues significantly decreases the likelihood of nurses encountering violence from these groups [8]. Caring‐oriented nursing practice and effective nurse–patient communication are important relational components of care that may improve patient trust, satisfaction, and mutual understanding [8–12]. However, from a resource‐based perspective, nurses’ perceived caring from themselves and their social and organizational environment is a psychosocial resource that supports compassionate care and helps nurses cope with demanding clinical situations [13, 14], such resources also need to be replenished constantly. Therefore, this study aimed to investigate surgical nurses’ perception of holistic humanistic caring and their experience of workplace violence and to examine the relationship between these two variables.

## 2. Background

Workplace violence constitutes a major occupational hazard on a global scale [1]. The International Labor Organization’s Violence and Harassment Convention, 2019 (No. 190), defines violence and harassment in the world of work as any act, action, or threat of harm within the work environment that results in physical, psychological, sexual, or economic harm, including gender‐based violence [15]. WPV inflicts significant harm on victims, with physical trauma being an evident adverse outcome. Furthermore, research suggests that WPV also causes profound psychological trauma, leading victims to experience feelings of isolation, vulnerability, insecurity, and job dissatisfaction [2, 16]. Victims often exhibit symptoms of anxiety, fear, and sleep disorders following such incidents [17].

Among healthcare professionals, nurses are particularly susceptible to WPV [1, 4, 18]. This heightened risk is attributed to their frequent direct contact with patients, coupled with factors such as insufficient safety training and the solitary nature of night shifts, which exacerbate their vulnerability to WPV [4]. Surgical nurses, in particular, face an elevated risk due to their responsibility for patients with specific needs, such as postoperative caring or those under the influence of medications like opioids [5, 6]. Empirical evidence from Sweden reveals that surgical nurses experience verbal violence at a rate of 28.8% and physical violence at 24.2% [5]. Similarly, a study conducted in Turkey reported WPV rates as high as 76% among surgical nurses [6].

Current WPV prevention strategies mainly focus on organizational, environmental, educational, and policy‐level interventions [1, 7, 19]. Systematic review evidence suggests that education, training, de‐escalation strategies, environmental safety measures, and organizational reporting mechanisms are commonly used to prevent or manage WPV in healthcare settings [7]. Therapeutic communication training has also been reported as a potentially useful intervention for reducing violence from patients against staff in clinical settings [9]. Few scholars have directed attention toward internal factors specific to nurses that could help prevent WPV incidents.

The concept of nurses’ holistic humanistic caring was proposed in 2023 by Yuqin Chen, a student of Chinese humanistic caring expert Yilan Liu [20]. This concept pertains to nurses’ perception of the caring they receive from both themselves and their surrounding social environment, which includes hospitals, departments, patients, colleagues, and friends. Such perceptions encapsulate the multidimensional support that nurses encounter in their professional and personal lives, thereby serving as a crucial metric for assessing their psychosocial resources. Empirical evidence suggests that integrating humanistic caring into nursing practice enhances patient satisfaction [10] and strengthens the trust between nurses and patients [21]. When nurses demonstrate respect towards patients and cultivate positive, trusting relationships, the incidence of WPV is likely to diminish [7]. Moreover, when nurses provide attentive caring and appropriate emotional support, patients often feel respected and understood, which can lead to a reduction in aggressive and violent behaviors [22, 23]. Engaging in proactive inquiries and maintaining open dialog as part of caring communication can alleviate patient dissatisfaction and misunderstandings, thereby reducing the risk of WPV [9]. Simultaneously, extending caring to colleagues in professional settings helps maintain daily collegial relationships.

According to Conservation of Resources theory, individuals strive to obtain, retain, protect, and replenish valued resources, and stress may occur when existing resources are depleted, threatened, or insufficiently replenished [13]. Resource replenishment is important for stress recovery, adaptation, and personal growth [24]. These resources may originate from internal personal factors, such as self‐esteem and resilience, as well as from external social systems, including social support and interpersonal relationships [13, 24]. From this theoretical perspective, caring can be regarded as an important psychosocial resource for nurses. Nurses with higher perceptions of holistic humanistic caring may have richer caring resources available from themselves, hospital managers, department coworkers, patients, family members, and friends. These accumulated caring resources may help nurses maintain sufficient emotional and psychological energy, enabling them to provide more caring‐oriented interactions with patients and colleagues while protecting their own well‐being. In turn, such caring‐oriented interactions may help reduce misunderstandings, interpersonal tension, and conflict in clinical settings, thereby theoretically lowering nurses’ risk of exposure to WPV [8, 9, 23].

The job demands–resources model also provides a theoretical basis for this assumption. This model proposes that adequate job and personal resources can buffer the negative effects of high job demands, such as occupational stress and emotional exhaustion [14]. In nursing contexts, occupational stress, emotional exhaustion, empathy, communication, and WPV have been shown to be interrelated [25–27]. Therefore, stronger holistic humanistic caring perception may serve as a multidimensional resource that helps nurses cope with demanding clinical situations, reduce emotional depletion, and maintain constructive communication with patients and colleagues.

However, although this theoretical reasoning is plausible, empirical evidence directly examining the association between nurses’ holistic humanistic caring perception and their experience of WPV remains limited. Therefore, this study aimed to provide empirical evidence for this theoretical inference by investigating the relationship between holistic humanistic caring perception and WPV among surgical nurses. This study aims to investigate the current perceptions of holistic humanistic caring among surgical nurses and the occurrence of WPV incidents, using data to demonstrate the correlation between them. It seeks to provide a scientific basis and practical guidance for enhancing nurses’ perceptions of holistic humanistic caring, thereby improving their professional fulfillment and well‐being, reducing occupational burnout, and optimizing the humanistic nursing environment. Additionally, it intends to offer both theoretical and empirical foundations for developing strategies that empower nurses to prevent and manage WPV from their own perspective, ultimately safeguarding their rights and interests.

## 3. Method

### 3.1. Design and Participants

In December 2024, surgical nurses from 30 tertiary and secondary general hospitals across 12 regions in Hubei Province were selected as research subjects using convenience sampling. Inclusion criteria are as follows: registered nurses holding a valid nursing practice certificate; informed consent and willingness to participate in this study. Exclusion criteria are as follows: those who had not engaged in relevant clinical nursing work for six months or more; individuals unable to directly participate in the survey. All subjects obtained informed consent and voluntarily participated in this study. The sample size was initially estimated using Kendall’s formula, which recommends a sample size of 5 to 10 times the number of variables [28]. Given that the questionnaire comprised 44 items, the initial minimum sample size was 220. Considering a potential nonresponse or invalid questionnaire rate of 20%, the minimum sample size was adjusted to 275 [29]. In addition, because the primary objective of this study was to examine the correlation between surgical nurses’ perception of holistic humanistic caring and their experience of WPV, an additional power analysis was conducted using G∗Power 3.1 [30]. For a two‐tailed correlation test, with a conservative small effect size of *r* = 0.10 based on Cohen’s criteria [31], *α* = 0.05, and power = 0.80, the required minimum sample size was approximately 783 participants. During the predefined data collection period, all eligible valid questionnaires were retained to improve statistical precision and avoid unnecessary exclusion of valid data. Ultimately, 937 questionnaires were collected, and 895 valid questionnaires were retained for analysis, exceeding the minimum sample size required for the primary correlation analysis.

### 3.2. Data Collection Instrument

The instruments used in this study included a self‐designed general information questionnaire, the Holistic Humanistic Caring Perception Scale for Nurses, and the Revised Hospital Workplace Violence Survey Questionnaire.

Through a preliminary literature review of observational studies on nurses’ holistic humanistic caring perception and WPV, and with reference to the STROBE recommendations for reporting observational studies, we designed a general information questionnaire tailored to the clinical practice context of surgical nursing [32, 33]. It primarily included gender, age, ethnicity, highest level of education, marital status, presence of children, cohabitation with family members, department, years of work, professional title, position, hospital grade, hospital category, hospital ownership, and one question: “Have you experienced any event in the past month that significantly impacted your emotional state?”

The Holistic Humanistic Caring Perception Scale for Nurses was developed by scholar Chen Yuqin in 2023, guided by role theory and caring theory [33]. It comprises six dimensions—self‐caring, hospital managers’ caring, department coworkers’ caring, patients’ caring, family caring, and friends’ caring—totaling 44 items. Each item is rated using a 5‐point Likert scale, ranging from “*never*” (1 point) to “*always*” (5 points). Higher scores on the overall scale and its dimensions indicate greater perceived humanistic caring among nurses. In this study, the Cronbach’s *α* coefficient for the entire scale was 0.983, with subscale coefficients of 0.929, 0.934, 0.987, 0.957, 0.974, and 0.983, respectively, indicating good reliability.

The Revised Hospital Workplace Violence Survey Questionnaire was developed by scholar Yang [34] based on the Hospital Workplace Violence Survey Questionnaire compiled by Chen et al. [35]. It comprises five sections: frequency of various types of WPV, description of experienced WPV incidents, individual perceptions and attitudes toward WPV, hospital policies and measures regarding WPV, and general demographic information. This study primarily utilized the questionnaire’s section on WPV frequency, specifically investigating nurses’ experiences of verbal abuse (verbal insults, verbal threats), physical assault, and sexual harassment over the past year. This section comprises four items, all multiple‐choice questions, and has been widely used in clinical settings. In this study, the Cronbach’s *α* coefficient for this section was 0.774, indicating good reliability (> 0.7).

### 3.3. Data Collection

After obtaining approval from the nursing supervisors of the respective departments, we conducted the online survey using a professional online questionnaire distribution and collection platform named “QuestionStar.” At the beginning of the questionnaire, we provided detailed explanations to respondents regarding the purpose of this study, completion requirements, and principles of informed consent and confidentiality. The collection of questionnaires was carried out on the premise of voluntary participation by respondents. Each respondent completed the questionnaire anonymously. The survey was designed to require completion of all questions before submission to ensure data integrity, and each IP address could submit only once.

A total of 937 questionnaire responses were collected. After data export, the research team conducted a quality control check before statistical analysis. Because all questionnaire items were set as mandatory in the online platform, no questionnaire contained missing values. Survey completion time was also reviewed as part of the data quality assessment. Given that the questionnaire included the informed consent statement, 15 demographic and work‐related questions, 44 items of the Holistic Humanistic Caring Perception Scale, and 4 items assessing WPV experience, completion in less than 180 s was considered unrealistically short. Questionnaires were considered invalid if they met one or more of the following criteria: (1) completion time of less than 180 s; (2) highly uniform responses across the scale items, such as selecting the same option for almost all Likert‐type items; (3) regular or wave‐like response patterns across consecutive items; or (4) obvious logical inconsistency in demographic or eligibility information [36]. Finally, 42 questionnaires were excluded, including 19 because of unrealistically short completion time, 12 because of highly uniform responses, 6 because of regular or wave‐like response patterns, and 5 because of logical inconsistency. When a questionnaire met more than one exclusion criterion, it was counted only once according to the primary reason for exclusion. A total of 895 valid questionnaires were retained for analysis, yielding a valid response rate of 95.52%.

### 3.4. Ethical Considerations

This study has been approved by the Ethics Committee of Tongji Medical College, Huazhong University of Science and Technology (approval no. S125) and has obtained the necessary permissions from the nursing directors of the participating hospitals. The questionnaire collection process was conducted on the premise of voluntary participation by the survey subjects.

### 3.5. Data Analysis

Data from the QuestionStar platform were downloaded in Excel format and subsequently subjected to a quality assurance process. Following verification, the data were imported into SPSS Version 26.0 for analysis. Descriptive statistics for the categorical data were presented as frequencies and percentages. The quantitative data were assessed for normality and found to be non‐normally distributed. Consequently, the median (M) along with the interquartile range (*P*_25_, *P*_75_) and the score rate (calculated as mean/total score × 100%) were employed to characterize the current status of each indicator. Univariate analyses were conducted using nonparametric tests, specifically the Mann–Whitney *U* test and the Kruskal–Wallis *H* test, to investigate demographic variables and identify differences. For variables with three or more groups, when the Kruskal–Wallis H test showed statistical significance, post hoc pairwise comparisons were further performed using Bonferroni correction. Only Bonferroni‐adjusted *p* values less than 0.05 were considered statistically significant in the post hoc analyses. Spearman’s rank correlation coefficient was utilized for correlation analysis. The associations between participants’ general characteristics and WPV experiences were examined using the chi‐square test or Fisher’s exact test, as appropriate. For this analysis, each type of WPV was dichotomized as “not experienced” and “experienced at least once” to improve interpretability and avoid sparse cells caused by the low occurrence of physical assault and sexual harassment. In addition, the original ordinal frequency categories of WPV were retained in the Spearman correlation analysis. A *p* value of less than 0.05 was considered indicative of statistical significance.

## 4. Results

### 4.1. Characteristics of the Participants

This study included 895 nurses aged 20 years to 57 years. The overall scores on the Holistic Humanistic Caring Perception Scale and scores across its dimensions did not follow a normal distribution for nurses with different demographic characteristics. Therefore, the data are presented using medians and quartiles, as detailed in Table 1.

**TABLE 1:** General characteristics and univariate analysis of factors associated with perceived holistic humanistic caring among surgical nurses (*N* = 895).

Variables/categories	*N* (%)	Self‐caring *M* (*P*_25_, *P*_75_)	Hospital managers’ caring *M* (*P*_25_, *P*_75_)	Department coworkers’ caring *M* (*P*_25_, *P*_75_)	Patients’ caring *M* (*P*_25_, *P*_75_)	Family caring *M* (*P*_25_, *P*_75_)	Friends’ caring *M* (*P*_25_, *P*_75_)	Total score *M* (*P*_25_, *P*_75_)
Gender								
Female	868 (96.98)	28.00 (26.00, 35.00)	16.00 (13.00, 20.00)	60.00 (55.00, 70.00)	12.00 (10.00, 15.00)	33.00 (28.00, 35.00)	20.00 (20.00, 25.00)	168.00 (152.00, 194.00)
Male	27 (3.02)	29.00 (21.00, 35.00)	16.00 (13.00, 20.00)	65.00 (56.00, 70.00)	12.00 (10.00, 15.00)	33.00 (25.00, 35.00)	24.00 (16.00, 25.00)	175.00 (140.00, 200.00)
*Z*/*H*	—	0.14	0.03	0.23	0.57	0.20	0.14	0.07
*p*	—	0.89	0.97	0.82	0.57	0.85	0.89	0.95
Age								
≤ 25	110 (12.29)	28.00 (26.00, 34.00)	16.00 (14.75, 20.00)	56.00 (55.00, 70.00)	12.00 (10.00, 15.00)	29.00 (27.00, 35.00)	20.00 (20.00, 25.00)	161.50 (151.75, 193.25)
26–30	198 (22.12)	30.50 (25.75, 35.00)	16.00 (12.00, 20.00)	60.50 (50.00, 70.00)	12.00 (9.00, 15.00)	35.00 (28.00, 35.00)	22.50 (20.00, 25.00)	170.00 (147.00, 197.50)
31–35	259 (28.94)	29.00 (25.00, 35.00)	16.00 (12.00, 20.00)	60.00 (53.00, 70.00)	12.00 (10.00, 15.00)	33.00 (28.00, 35.00)	20.00 (20.00, 25.00)	168.00 (148.00, 196.00)
36–40	176 (19.66)	28.00 (26.00, 34.00)	16.00 (13.00, 20.00)	60.00 (55.00, 70.00)	12.00 (10.00, 15.00)	34.00 (28.00, 35.00)	20.00 (19.00, 25.00)	171.00 (153.25, 192.00)
41–45	88 (9.83)	28.00 (25.00, 34.75)	16.00 (13.00, 20.00)	60.50 (55.00, 70.00)	12.00 (11.00, 14.75)	30.50 (28.00, 35.00)	20.00 (20.00, 25.00)	165.50 (152.25, 190.50)
> 45	64 (7.15)	29.00 (26.00, 32.00)	17.00 (14.00, 19.75)	61.50 (56.00, 70.00)	12.00 (10.00, 14.75)	32.00 (27.00, 35.00)	21.00 (18.25, 25.00)	172.00 (154.00, 189.50)
*Z*/*H*	—	6.56	4.07	0.66	1.24	4.20	2.66	0.89
*p*	—	0.26	0.54	0.99	0.94	0.52	0.75	0.97
Ethnicity								
Chinese Han population	780 (87.15)	29.00 (26.00, 35.00)	16.00 (13.00, 20.00)	61.00 (55.25, 70.00)	12.00 (10.00, 15.00)	33.00 (28.00, 35.00)	20.00 (20.00, 25.00)	169.50 (153.00, 194.00)
Chinese national minority	115 (12.85)	28.00 (22.00, 34.00)	14.00 (12.00, 20.00)	56.00 (45.00, 70.00)	12.00 (9.00, 15.00)	32.00 (26.00, 35.00)	20.00 (16.00, 25.00)	160.00 (136.00, 193.00)
*Z*/*H*	—	2.31	3.19	2.49	2.62	1.13	1.88	2.59
*p*	—	**< 0.05**	**< 0.05**	**< 0.05**	**< 0.05**	0.26	0.06	**< 0.05**
Highest academic degree obtained								
College degree	61 (6.82)	28.00 (22.50, 35.00)	16.00 (12.00, 20.00)	57.00 (51.50, 70.00)	12.00 (9.00, 14.50)	29.00 (24.00, 35.00)	20.00 (19.00, 25.00)	163.00 (139.50, 193.50)
Bachelor degree	812 (90.73)	28.00 (26.00, 35.00)	16.00 (13.00, 20.00)	60.00 (55.00, 70.00)	12.00 (10.00, 15.00)	33.00 (28.00, 35.00)	20.00 (20.00, 25.00)	168.50 (153.00, 194.75)
Master degree or above	22 (2.46)	29.50 (21.75, 33.50)	16.00 (12.00, 18.00)	56.00 (51.75, 69.25)	11.50 (9.00, 12.00)	28.00 (25.25, 33.25)	20.00 (15.00, 24.25)	163.00 (130.75, 182.00)
*Z*/*H*	—	0.95	1.36	1.13	4.97	7.52	3.87	2.16
*p*	—	0.62	0.51	0.29	0.08	**< 0.05**	0.14	0.34
Marital status								
Unmarried	229 (25.59)	28.00 (25.00, 35.00)	16.00 (13.00, 20.00)	56.00 (54.00, 70.00)	12.00 (9.00, 15.00)	29.00 (27.50, 35.00)	20.00 (20.00, 25.00)	163.00 (149.00, 193.00)
Married	650 (72.63)	29.00 (26.00, 35.00)	16.00 (13.00, 20.00)	61.00 (55.00, 70.00)	12.00 (10.00, 15.00)	33.00 (28.00, 35.00)	20.00 (20.00, 25.00)	170.00 (152.75, 195.00)
Divorced	16 (1.79)	29.50 (25.25, 35.00)	16.00 (14.25, 20.00)	67.00 (56.50, 70.00)	12.00 (9.75, 15.00)	31.50 (26.50, 35.00)	20.00 (18.25, 25.00)	176.50 (161.50, 198.00)
*Z*/*H*	—	0.39	0.26	3.51	6.08	3.71	0.11	2.95
*p*	—	0.82	0.88	0.17	**< 0.05**	0.16	0.95	0.23
Having children								
No	306 (34.19)	28.00 (26.00, 35.00)	16.00 (13.00, 20.00)	59.00 (54.00, 70.00)	12.00 (9.00, 15.00)	32.00 (28.00, 35.00)	20.00 (20.00, 25.00)	167.00 (151.00, 195.00)
Yes	589 (65.81)	28.00 (25.50, 35.00)	16.00 (13.00, 20.00)	60.00 (55.00, 70.00)	12.00 (10.00, 15.00)	33.00 (28.00, 35.00)	20.00 (19.00, 25.00)	169.00 (152.00, 193.00)
*Z*/*H*	—	0.68	1.12	0.36	1.66	0.20	0.83	0.01
*p*	—	0.50	0.26	0.72	0.10	0.85	0.41	0.99
Living with family								
No	106 (11.84)	28.00 (25.00, 35.00)	16.00 (12.75, 20.00)	56.00 (49.75, 70.00)	12.00 (9.00, 12.25)	28.00 (26.00, 35.00)	20.00 (18.75, 25.00)	160.00 (147.00, 187.00)
Yes	789 (88.16)	29.00 (26.00, 35.00)	16.00 (13.00, 20.00)	61.00 (55.00, 70.00)	12.00 (10.00, 15.00)	33.00 (28.00, 35.00)	20.00 (20.00, 25.00)	170.00 (152.00, 195.00)
*Z*/*H*	—	0.31	0.62	2.54	3.07	2.47	2.00	2.28
*p*	—	0.76	0.54	**< 0.05**	**< 0.05**	**< 0.05**	**< 0.05**	**< 0.05**
Department								
Gastrointestinal surgery	181 (20.22)	29.00 (25.00, 35.00)	16.00 (12.00, 20.00)	59.00 (54.00, 70.00)	12.00 (10.00, 15.00)	33.00 (28.00, 35.00)	20.00 (20.00, 25.00)	167.00 (147.50, 195.00)
Hepatobiliary surgery	76 (8.49)	28.00 (24.25, 35.00)	16.00 (12.25, 20.00)	59.00 (48.00, 70.00)	12.00 (9.00, 15.00)	29.50 (24.50, 35.00)	20.00 (15.00, 25.00)	165.50 (135.25, 196.75)
Thyroid and Breast Surgery	141 (15.75)	29.00 (26.00, 35.00)	16.00 (13.50, 20.00)	62.00 (55.00, 70.00)	12.00 (12.00, 15.00)	34.00 (28.00, 35.00)	21.00 (20.00, 25.00)	175.00 (154.50, 193.00)
Thoracic surgery	45 (5.03)	27.00 (24.00, 31.00)	15.00 (12.00, 20.00)	56.00 (47.00, 70.00)	12.00 (9.00, 12.00)	28.00 (22.50, 35.00)	20.00 (16.00, 24.50)	160.00 (127.50, 184.50)
Urology	41 (4.58)	28.00 (24.50, 34.00)	16.00 (12.50, 20.00)	59.00 (56.00, 70.00)	12.00 (11.00, 15.00)	34.00 (28.00, 35.00)	25.00 (20.00, 25.00)	171.00 (154.50, 193.50)
Pancreatic surgery	15 (1.68)	28.00 (27.00, 35.00)	16.00 (16.00, 20.00)	70.00 (70.00, 70.00)	12.00 (9.00, 15.00)	28.00 (25.00, 35.00)	20.00 (20.00, 25.00)	175.00 (161.00, 196.00)
Cardiac surgery	5 (0.56)	21.00 (21.00, 34.00)	12.00 (11.00, 17.50)	42.00 (41.50, 69.00)	9.00 (7.50, 13.50)	28.00 (21.00, 35.00)	20.00 (12.50, 25.00)	125.00 (118.00, 194.00)
Neurosurgery	40 (4.47)	28.00 (24.00, 34.50)	15.00 (10.50, 19.75)	56.00 (47.50, 70.00)	12.00 (9.00, 15.00)	28.00 (25.25, 35.00)	20.00 (20.00, 25.00)	160.50 (134.25, 190.25)
Vascular surgery	19 (2.12)	28.00 (26.00, 35.00)	16.00 (12.00, 20.00)	59.00 (51.00, 70.00)	12.00 (11.00, 15.00)	35.00 (28.00, 35.00)	23.00 (20.00, 25.00)	171.00 (146.00, 196.00)
Orthopedic surgery	135 (15.08)	29.00 (26.00, 35.00)	16.00 (15.00, 20.00)	64.00 (56.00, 70.00)	12.00 (10.00, 15.00)	35.00 (28.00, 35.00)	22.00 (20.00, 25.00)	173.00 (155.00, 194.00)
Hand surgery	24 (2.68)	34.50 (29.25, 35.00)	20.00 (18.25, 20.00)	70.00 (70.00, 70.00)	15.00 (12.00, 15.00)	35.00 (35.00, 35.00)	25.00 (25.00, 25.00)	195.50 (178.75, 200.00)
Gynecology	16 (1.79)	28.00 (23.00, 29.00)	16.00 (14.00, 18.25)	58.00 (56.00, 70.00)	12.00 (10.25, 13.75)	28.50 (24.75, 34.00)	20.00 (19.25, 25.00)	169.50 (152.25, 183.50)
Otolaryngology—head and neck surgery	11 (1.23)	32.00 (28.00, 35.00)	15.00 (12.00, 20.00)	69.00 (47.00, 70.00)	12.00 (9.00, 15.00)	35.00 (28.00, 35.00)	21.00 (15.00, 25.00)	167.00 (145.00, 197.00)
Pediatric surgery	21 (2.35)	32.00 (26.00, 35.00)	17.00 (13.50, 20.00)	67.00 (55.50, 70.00)	12.00 (9.50, 15.00)	35.00 (28.00, 35.00)	25.00 (20.00, 25.00)	185.00 (157.00, 199.00)
General surgery	125 (13.97)	28.00 (25.50, 35.00)	16.00 (12.50, 20.00)	56.00 (54.00, 70.00)	12.00 (9.00, 15.00)	30.00 (27.50, 35.00)	20.00 (18.50, 25.00)	160.00 (151.50, 191.00)
*Z*/*H*	—	28.98	39.88	43.05	31.84	35.78	35.39	36.97
*p*	—	**< 0.05**	**< 0.05**	**< 0.05**	**< 0.05**	**< 0.05**	**< 0.05**	**< 0.05**
Years of work								
< 3	107 (11.96)	28.00 (26.00, 35.00)	16.00 (15.00, 20.00)	61.00 (56.00, 70.00)	12.00 (9.00, 15.00)	30.00 (27.00, 35.00)	20.00 (20.00, 25.00)	164.00 (153.00, 196.00)
3–5	115 (12.85)	29.00 (26.00, 35.00)	16.00 (12.00, 20.00)	62.00 (51.00, 70.00)	12.00 (9.00, 15.00)	34.00 (28.00, 35.00)	25.00 (20.00, 25.00)	172.00 (149.00, 196.00)
6–10	213 (23.80)	29.00 (25.00, 34.50)	16.00 (12.00, 20.00)	56.00 (51.50, 70.00)	12.00 (10.00, 15.00)	32.00 (28.00, 35.00)	20.00 (20.00, 25.00)	163.00 (146.50, 192.00)
11–15	240 (26.82)	28.00 (25.25, 35.00)	16.00 (12.00, 20.00)	66.00 (56.00, 70.00)	12.00 (10.00, 15.00)	35.00 (28.00, 35.00)	20.50 (20.00, 25.00)	173.00 (153.25, 197.00)
16–20	111 (12.40)	28.00 (25.00, 34.00)	16.00 (13.00, 20.00)	60.00 (55.00, 70.00)	12.00 (11.00, 15.00)	34.00 (28.00, 35.00)	20.00 (19.00, 25.00)	168.00 (152.00, 192.00)
> 20	109 (12.18)	28.00 (26.00, 32.00)	16.00 (13.00, 19.50)	60.00 (55.00, 70.00)	12.00 (9.50, 14.00)	31.00 (28.00, 35.00)	20.00 (19.00, 25.00)	167.00 (150.00, 187.00)
*Z*/*H*	—	3.10	7.83	6.92	5.77	4.26	3.59	3.50
*p*	—	0.69	0.17	0.23	0.33	0.51	0.61	0.62
Professional title								
Nurse	119 (13.30)	28.00 (25.00, 35.00)	16.00 (15.00, 20.00)	59.00 (55.00, 70.00)	12.00 (9.00, 15.00)	30.00 (27.00, 35.00)	20.00 (20.00, 25.00)	164.00 (150.00, 195.00)
Senior nurse	288 (32.18)	29.00 (25.00, 35.00)	16.00 (12.00, 20.00)	61.50 (53.25, 70.00)	12.00 (9.00, 15.00)	34.00 (28.00, 35.00)	21.00 (20.00, 25.00)	168.50 (149.25, 196.00)
Supervisor nurse	438 (48.94)	28.00 (26.00, 34.25)	16.00 (12.00, 20.00)	59.00 (54.00, 70.00)	12.00 (10.00, 15.00)	33.00 (28.00, 35.00)	20.00 (20.00, 25.00)	169.00 (152.00, 193.00)
Deputy director nurse	47 (5.25)	29.00 (25.00, 32.00)	16.00 (13.00, 19.00)	59.00 (56.00, 69.00)	12.00 (10.00, 12.00)	31.00 (28.00, 35.00)	20.00 (19.00, 25.00)	166.00 (152.00, 188.00)
Director nurse	3 (0.34)	28.00 (28.00, —)	18.00 (17.00, —)	65.00 (65.00, —)	15.00 (14.00, —)	32.00 (32.00, —)	24.00 (24.00, —)	181.00 (181.00, —)
*Z*/*H*	—	1.52	8.16	0.91	5.62	2.43	2.25	2.23
*p*	—	0.82	0.09	0.92	0.23	0.66	0.69	0.69
Position								
Nurse	819 (91.51)	28.00 (26.00, 35.00)	16.00 (13.00, 20.00)	60.00 (54.00, 70.00)	12.00 (10.00, 15.00)	33.00 (28.00, 35.00)	20.00 (20.00, 25.00)	168.00 (151.00, 195.00)
Head nurse	76 (8.49)	28.00 (25.00, 32.00)	16.00 (13.00, 19.75)	62.00 (56.00, 70.00)	12.00 (10.00, 13.00)	32.00 (28.00, 35.00)	20.00 (19.25, 25.00)	171.50 (152.00, 188.00)
*Z*/*H*	—	1.83	0.78	0.33	0.90	0.22	0.25	0.45
*p*	—	0.07	0.43	0.74	0.37	0.82	0.80	0.65
Hospital level								
Tertiary hospital	837 (93.52)	29.00 (26.00, 35.00)	16.00 (13.00, 20.00)	61.00 (55.00, 70.00)	12.00 (10.00, 15.00)	33.00 (28.00, 35.00)	20.00 (20.00, 25.00)	169.00 (153.00, 194.00)
Secondary hospital	58 (6.48)	25.50 (21.00, 32.25)	12.50 (11.75, 20.00)	56.00 (42.00, 70.00)	12.00 (9.00, 14.25)	28.00 (21.00, 35.00)	20.00 (15.00, 25.00)	160.00 (120.00, 188.00)
*Z*/*H*	—	3.31	3.14	2.77	2.57	2.47	2.45	2.80
*p*	—	**< 0.05**	**< 0.05**	**< 0.05**	**< 0.05**	**< 0.05**	**< 0.05**	**< 0.05**
Hospital category								
General hospital	876 (97.88)	28.00 (26.00, 35.00)	16.00 (13.00, 20.00)	60.00 (55.00, 70.00)	12.00 (10.00, 15.00)	33.00 (28.00, 35.00)	20.00 (20.00, 25.00)	168.50 (151.25, 194.00)
Specialized hospital	19 (2.12)	27.00 (21.00, 34.00)	14.00 (11.00, 18.00)	56.00 (50.00, 70.00)	12.00 (9.00, 14.00)	28.00 (26.00, 35.00)	20.00 (15.00, 25.00)	161.00 (137.00, 184.00)
*Z*/*H*	—	1.26	1.66	0.69	0.42	1.22	1.70	1.46
*p*	—	0.21	0.10	0.49	0.68	0.22	0.09	0.15
Hospital ownership								
Governmental hospital	882 (98.55)	28.50 (26.00, 35.00)	16.00 (13.00, 20.00)	60.00 (55.00, 70.00)	12.00 (10.00, 15.00)	33.00 (28.00, 35.00)	20.00 (20.00, 25.00)	168.50 (152.00, 194.25)
Private hospital	13 (1.45)	26.00 (22.50, 28.50)	13.00 (11.50, 16.00)	56.00 (49.00, 56.00)	11.00 (9.50, 12.00)	28.00 (25.50, 31.50)	20.00 (18.00, 23.50)	147.00 (137.00, 167.50)
*Z*/*H*	—	2.17	2.32	2.65	1.71	1.90	0.99	2.43
*p*	—	**< 0.05**	**< 0.05**	**< 0.05**	0.09	0.06	0.33	**< 0.05**
Recent event with significant emotional impact								
No	797 (89.05)	29.00 (26.00, 35.00)	16.00 (13.00, 20.00)	60.00 (55.00, 70.00)	12.00 (10.00, 15.00)	33.00 (28.00, 35.00)	20.00 (20.00, 25.00)	168.00 (153.00, 195.00)
Yes, negative impact	47 (5.25)	24.00 (21.00, 29.00)	12.00 (11.00, 17.00)	50.00 (42.00, 66.00)	11.00 (9.00, 15.00)	28.00 (21.00, 35.00)	20.00 (15.00, 25.00)	145.00 (124.00, 180.00)
Yes, positive impact	51 (5.70)	29.00 (25.00, 35.00)	16.00 (12.00, 20.00)	69.00 (56.00, 70.00)	12.00 (9.00, 15.00)	35.00 (28.00, 35.00)	20.00 (20.00, 25.00)	178.00 (158.00, 200.00)
*Z*/*H*	—	21.84	13.34	17.64	5.32	6.59	2.23	18.42
*p*	—	**< 0.05**	**< 0.05**	**< 0.05**	0.07	**< 0.05**	0.33	**< 0.05**

*Note:* Data are presented as median and interquartile range, *M* (*P*_25_, *P*_75_), unless otherwise indicated. Mann–Whitney *U* tests were used for variables with two groups, and Kruskal–Wallis *H* tests were used for variables with three or more groups. *Z* values are reported for Mann–Whitney *U* tests, and *H* values are reported for Kruskal–Wallis *H* tests. “—” indicates not applicable. For the director nurse subgroup, *P*_75_ was not calculated by SPSS because of the very small subgroup size (*n* = 3). *p* < 0.05 was considered statistically significant. Bold *p* values indicate *p* < 0.05.

### 4.2. Current Status of Surgical Nurses’ Perception of Holistic Humanistic Caring

The scores on the Holistic Humanistic Caring Perception Scale for 895 surgical nurses, as well as scores across its dimensions, did not follow a normal distribution. Therefore, the median and quartiles were used to present the scale scores. The median and quartiles for the total score on the Holistic Humanistic Caring Perception Scale among the 895 surgical nurses were 168.00 (151.00, 194.00). The median and interquartile ranges for the six dimensions—self‐caring, hospital managers’ caring, department coworkers’ caring, patients’ caring, family caring, and friends’ caring—are shown in Table 2, with score rates of 82.46%, 79.20%, 84.83%, 80.27%, 86.43%, and 84.56%, respectively. Surgical nurses perceived the lowest level of caring from hospital managers and the highest level of caring from family members.

**TABLE 2:** Current status of surgical nurses’ perception of holistic humanistic caring.

Variables	*M* (*P*_25_, *P*_75_)	Mean ± SD	Top‐scoring items	Lowest‐scoring items
Self‐caring	28.00 (26.00, 35.00)	28.86 ± 5.41	I’m happy to be able to help others.	I don’t put too much pressure on myself. When needed, I actively seek help from others, such as family, colleagues, friends, or professional counseling.
Hospital managers’ caring	16.00 (13.00, 20.00)	15.84 ± 3.76	The hospital managers guarantee nurses’ statutory benefits (such as holiday rotation schedules)	The hospital has a mechanism for nurses to participate in decision‐making.
Department coworkers’ caring	60.00 (55.00, 70.00)	59.38 ± 11.28	Colleagues proactively offer assistance when I’m facing difficulties or feeling unwell.	The department takes the initiative to show concern for nurses and actively provides whatever assistance it can when nurses face difficulties (such as family conflicts).
Patients’ caring	12.00 (10.00, 15.00)	12.04 ± 2.52	The patients or their family member expressed understanding regarding the flaws in my work.	Patients or their families have commended my work and encouraged me.
Family caring	33.00 (28.00, 35.00)	30.25 ± 5.58	My family members are concerned about my health.	When I’m with my family, they comfort me through physical contact, such as hugs.
Friends’ caring	20.00 (20.00, 25.00)	21.14 ± 4.08	We are tolerant and accommodating of each other./Friends will actively support and help me whenever I need it.	Friends show me caring through material gestures (such as small gifts, drinks, etc.).

### 4.3. Single‐Factor Analysis of Surgical Nurses’ Perception of Holistic Humanistic Caring

Univariate analysis revealed that ethnicity was associated with self‐caring, hospital managers’ caring, department coworkers’ caring, patients’ caring, and the total score; highest academic degree was associated with family caring; marital status was associated with patients’ caring; and living with family was associated with department coworkers’ caring, patients’ caring, family caring, friends’ caring, and the total score. Hospital ownership was associated with self‐caring, hospital managers’ caring, department coworkers’ caring, and the total score, while department and hospital level were associated with all caring dimensions and the total score. Recent emotionally influential events were associated with self‐caring, hospital managers’ caring, department coworkers’ caring, family caring, and the total score (see Table 1).

Post hoc pairwise comparisons with Bonferroni correction were further performed for variables with three or more groups when the Kruskal–Wallis H test showed statistical significance. The post hoc results showed that significant pairwise differences were mainly observed across departments and recent emotionally influential events. In terms of department, nurses in hand surgery generally had higher mean ranks than those in several other departments for hospital managers’ caring, department coworkers’ caring, patients’ caring, friends’ caring, and the total score. Nurses in pancreatic surgery also had higher mean ranks for department coworkers’ caring than nurses in thoracic surgery, neurosurgery, and general surgery. In contrast, thoracic surgery nurses had lower mean ranks for family caring than nurses in orthopedic surgery and hand surgery. Regarding recent emotionally influential events, nurses who had experienced negative events had lower mean ranks for hospital managers’ caring than those who had not experienced such events or those who had experienced positive events. Detailed significant pairwise comparisons are presented in Table 3.

**TABLE 3:** Significant post hoc pairwise comparisons after Kruskal–Wallis *H* tests.

Variable	Dimension	Group 1	Mean rank 1	Group 2	Mean rank 2	Higher mean‐rank group	Adjusted *p*
Department	Hospital managers’ caring	Gastrointestinal surgery	423.60	Hand surgery	665.29	Hand surgery	0.001
		Hepatobiliary surgery	450.05	Hand surgery	665.29	Hand surgery	0.029
		Thyroid and breast surgery	469.85	Hand surgery	665.29	Hand surgery	0.049
		Thoracic surgery	376.28	Hand surgery	665.29	Hand surgery	< 0.001
		Urology	432.77	Hand surgery	665.29	Hand surgery	0.036
		Neurosurgery	370.91	Hand surgery	665.29	Hand surgery	< 0.001
		Hand surgery	665.29	General surgery	421.95	Hand surgery	0.002
	Department coworkers’ caring	Gastrointestinal surgery	443.39	Hand surgery	655.60	Hand surgery	0.011
		Hepatobiliary surgery	427.71	Hand surgery	655.60	Hand surgery	0.011
		Thyroid and breast surgery	456.48	Hand surgery	655.60	Hand surgery	0.035
		Thoracic surgery	392.91	Pancreatic surgery	661.87	Pancreatic surgery	0.035
		Thoracic surgery	392.91	Hand surgery	655.60	Hand surgery	0.004
		Pancreatic surgery	661.87	Neurosurgery	368.31	Pancreatic surgery	0.012
		Pancreatic surgery	661.87	General surgery	403.92	Pancreatic surgery	0.018
		Neurosurgery	368.31	Hand surgery	655.60	Hand surgery	0.001
		Hand surgery	655.60	General surgery	403.92	Hand surgery	< 0.001
	Patients’ caring	Thoracic surgery	355.09	Hand surgery	623.21	Hand surgery	0.002
		Hand surgery	623.21	General surgery	415.82	Hand surgery	0.021
	Family caring	Thoracic surgery	330.64	Orthopedic surgery	487.55	Orthopedic surgery	0.021
		Thoracic surgery	330.64	Hand surgery	590.10	Hand surgery	0.003
	Friends’ caring	Hepatobiliary surgery	397.91	Hand surgery	628.90	Hand surgery	0.006
		Thoracic surgery	340.49	Hand surgery	628.90	Hand surgery	< 0.001
		Neurosurgery	404.63	Hand surgery	628.90	Hand surgery	0.042
		Hand surgery	628.90	General surgery	411.88	Hand surgery	0.007
	Total score	Gastrointestinal surgery	445.69	Hand surgery	669.90	Hand surgery	0.007
		Hepatobiliary surgery	422.95	Hand surgery	669.90	Hand surgery	0.005
		Thyroid and breast surgery	468.15	Hand surgery	669.90	Hand surgery	0.041
		Thoracic surgery	343.33	Hand surgery	669.90	Hand surgery	< 0.001
		Neurosurgery	391.90	Hand surgery	669.90	Hand surgery	0.003
		Hand surgery	669.90	General surgery	413.27	Hand surgery	< 0.001

Recent event with significant emotional impact	Hospital managers’ caring	No	453.80	Yes, negative impact	318.80	No	0.001
		Yes, negative impact	318.80	Yes, positive impact	476.40	Yes, positive impact	0.006

*Note:* Only statistically significant pairwise comparisons (Bonferroni‐adjusted *p* < 0.05) are shown. Pairwise comparisons were performed following significant Kruskal–Wallis *H* tests. Adjusted *p* values were generated by SPSS with Bonferroni correction. Higher mean ranks indicate higher perceived holistic humanistic caring scores in the corresponding dimension. No Bonferroni‐adjusted pairwise comparison reached statistical significance for highest academic degree in family caring or marital status in patients’ caring.

### 4.4. Current Status of Surgical Nurses Experiencing WPV

The current status of WPV experienced by the 895 surgical nurses participating in the survey is shown in Table 4. Among the various types of WPV, verbal abuse was the most frequently reported form, followed by verbal threats, physical assault, and sexual harassment.

**TABLE 4:** Current status of surgical nurses experiencing WPV (*N* = 895, *n* (%)).

Number of times exposed to WPV	Verbal violence		Physical assault	Sexual harassment
	Verbal abuse	Verbal threats		
0	667 (74.53)	761 (85.03)	856 (95.64)	866 (96.76)
1	135 (15.08)	76 (8.49)	20 (2.23)	16 (1.79)
2‐3	55 (6.15)	35 (3.91)	13 (1.45)	9 (1.01)
> 3	38 (4.25)	23 (2.57)	6 (0.67)	4 (0.45)

In addition to describing the overall occurrence of WPV, we further examined differences in WPV experiences according to participants’ general characteristics. Each type of WPV was dichotomized as “not experienced” and “experienced at least once.” The results showed that physical assault differed significantly by gender, marital status, having children, years of work, professional title, hospital level, and hospital category. Sexual harassment differed significantly by gender and hospital level. Verbal abuse differed significantly by highest academic degree obtained and recent emotionally influential events, while verbal threats differed significantly by recent emotionally influential events. Detailed results are presented in Table 5.

**TABLE 5:** Differences in workplace violence experiences according to participants’ general characteristics (*N* = 895).

Characteristics/categories	*N* (%)	Verbal abuse ≥ 1 *N* (%)	*p*	Verbal threats ≥ 1 *N* (%)	*p*	Physical assault ≥ 1 *N* (%)	*p*	Sexual harassment ≥ 1 *N* (%)	*p*
Gender									
Female	868 (96.98)	219 (25.23)	0.341	127 (14.63)	0.164	35 (4.03)	**0.026**	25 (2.88)	**0.009**
Male	27 (3.02)	9 (33.33)	0.341	7 (25.93)	0.164	4 (14.81)	**0.026**	4 (14.81)	**0.009**
Age									
≤ 25	110 (12.29)	23 (20.91)	0.785	16 (14.55)	0.226	8 (7.27)	0.124	5 (4.55)	0.731
26–30	198 (22.12)	51 (25.76)	0.785	23 (11.62)	0.226	13 (6.57)	0.124	7 (3.54)	0.731
31–35	259 (28.94)	65 (25.10)	0.785	43 (16.60)	0.226	8 (3.09)	0.124	10 (3.86)	0.731
36–40	176 (19.66)	48 (27.27)	0.785	27 (15.34)	0.226	8 (4.55)	0.124	3 (1.70)	0.731
41–45	88 (9.83)	26 (29.55)	0.785	19 (21.59)	0.226	1 (1.14)	0.124	3 (3.41)	0.731
> 45	64 (7.15)	15 (23.44)	0.785	6 (9.38)	0.226	1 (1.56)	0.124	1 (1.56)	0.731
Ethnicity									
Chinese Han population	780 (87.15)	199 (25.51)	0.946	117 (15.00)	0.951	31 (3.97)	0.144	24 (3.08)	0.406
Chinese national minority	115 (12.85)	29 (25.22)	0.946	17 (14.78)	0.951	8 (6.96)	0.144	5 (4.35)	0.406
Highest academic degree obtained									
College degree	61 (6.82)	7 (11.48)	**0.019**	6 (9.84)	0.144	3 (4.92)	0.722	4 (6.56)	0.153
Bachelor degree	812 (90.73)	213 (26.23)	**0.019**	122 (15.02)	0.144	36 (4.43)	0.722	24 (2.96)	0.153
Master degree or above	22 (2.46)	8 (36.36)	**0.019**	6 (27.27)	0.144	0 (0.00)	0.722	1 (4.55)	0.153
Marital status									
Unmarried	229 (25.59)	58 (25.33)	0.819	34 (14.85)	0.958	18 (7.86)	**0.009**	11 (4.80)	0.249
Married	650 (72.63)	167 (25.69)	0.819	98 (15.08)	0.958	21 (3.23)	**0.009**	18 (2.77)	0.249
Divorced	16 (1.79)	3 (18.75)	0.819	2 (12.50)	0.958	0 (0.00)	**0.009**	0 (0.00)	0.249
Having children									
No	306 (34.19)	73 (23.86)	0.423	39 (12.75)	0.178	21 (6.86)	**0.008**	13 (4.25)	0.220
Yes	589 (65.81)	155 (26.32)	0.423	95 (16.13)	0.178	18 (3.06)	**0.008**	16 (2.72)	0.220
Living with family									
No	106 (11.84)	32 (30.19)	0.236	14 (13.21)	0.588	8 (7.55)	0.121	2 (1.89)	0.564
Yes	789 (88.16)	196 (24.84)	0.236	120 (15.21)	0.588	31 (3.93)	0.121	27 (3.42)	0.564
Department									
Gastrointestinal surgery	181 (20.22)	36 (19.89)	0.076	21 (11.60)	0.773	4 (2.21)	0.398	5 (2.76)	0.824
Hepatobiliary surgery	76 (8.49)	15 (19.74)	0.076	11 (14.47)	0.773	4 (5.26)	0.398	4 (5.26)	0.824
Thyroid and breast surgery	141 (15.75)	35 (24.82)	0.076	19 (13.48)	0.773	6 (4.26)	0.398	3 (2.13)	0.824
Thoracic surgery	45 (5.03)	12 (26.67)	0.076	6 (13.33)	0.773	2 (4.44)	0.398	2 (4.44)	0.824
Urology	41 (4.58)	8 (19.51)	0.076	7 (17.07)	0.773	1 (2.44)	0.398	1 (2.44)	0.824
Pancreatic surgery	15 (1.68)	5 (33.33)	0.076	1 (6.67)	0.773	0 (0.00)	0.398	0 (0.00)	0.824
Cardiac surgery	5 (0.56)	3 (60.00)	0.076	1 (20.00)	0.773	0 (0.00)	0.398	0 (0.00)	0.824
Neurosurgery	40 (4.47)	7 (17.50)	0.076	7 (17.50)	0.773	2 (5.00)	0.398	3 (7.50)	0.824
Vascular surgery	19 (2.12)	8 (42.11)	0.076	3 (15.79)	0.773	0 (0.00)	0.398	0 (0.00)	0.824
Orthopedic surgery	135 (15.08)	46 (34.07)	0.076	24 (17.78)	0.773	4 (2.96)	0.398	3 (2.22)	0.824
Hand surgery	24 (2.68)	4 (16.67)	0.076	1 (4.17)	0.773	1 (4.17)	0.398	1 (4.17)	0.824
Gynecology	16 (1.79)	3 (18.75)	0.076	2 (12.50)	0.773	1 (6.25)	0.398	0 (0.00)	0.824
Otolaryngology—head and neck surgery	11 (1.23)	4 (36.36)	0.076	2 (18.18)	0.773	0 (0.00)	0.398	0 (0.00)	0.824
Pediatric surgery	21 (2.35)	8 (38.10)	0.076	4 (19.05)	0.773	2 (9.52)	0.398	0 (0.00)	0.824
General surgery	125 (13.97)	34 (27.20)	0.076	25 (20.00)	0.773	12 (9.60)	0.398	7 (5.60)	0.824
Years of work									
< 3	107 (11.96)	20 (18.69)	0.527	14 (13.08)	0.553	6 (5.61)	**0.005**	3 (2.80)	0.383
3–5	115 (12.85)	33 (28.70)	0.527	17 (14.78)	0.553	13 (11.30)	**0.005**	8 (6.96)	0.383
6–10	213 (23.80)	52 (24.41)	0.527	26 (12.21)	0.553	7 (3.29)	**0.005**	7 (3.29)	0.383
11–15	240 (26.82)	67 (27.92)	0.527	44 (18.33)	0.553	10 (4.17)	**0.005**	6 (2.50)	0.383
16–20	111 (12.40)	29 (26.13)	0.527	18 (16.22)	0.553	2 (1.80)	**0.005**	3 (2.70)	0.383
> 20	109 (12.18)	27 (24.77)	0.527	15 (13.76)	0.553	1 (0.92)	**0.005**	2 (1.83)	0.383
Professional title									
Nurse	119 (13.30)	25 (21.01)	0.415	18 (15.13)	0.932	9 (7.56)	**0.013**	5 (4.20)	0.476
Senior nurse	288 (32.18)	69 (23.96)	0.415	41 (14.24)	0.932	19 (6.60)	**0.013**	12 (4.17)	0.476
Supervisor nurse	438 (48.94)	122 (27.85)	0.415	67 (15.30)	0.932	11 (2.51)	**0.013**	12 (2.74)	0.476
Deputy director nurse	47 (5.25)	12 (25.53)	0.415	8 (17.02)	0.932	0 (0.00)	**0.013**	0 (0.00)	0.476
Director nurse	3 (0.34)	0 (0.00)	0.415	0 (0.00)	0.932	0 (0.00)	**0.013**	0 (0.00)	0.476
Position									
Nurse	819 (91.51)	206 (25.15)	0.468	118 (14.41)	0.120	39 (4.76)	0.069	29 (3.54)	0.164
Head nurse	76 (8.49)	22 (28.95)	0.468	16 (21.05)	0.120	0 (0.00)	0.069	0 (0.00)	0.164
Hospital level									
Tertiary hospital	837 (93.52)	213 (25.45)	0.944	122 (14.58)	0.207	33 (3.94)	**0.035**	24 (2.87)	**0.034**
Secondary hospital	58 (6.48)	15 (25.86)	0.944	12 (20.69)	0.207	6 (10.34)	**0.035**	5 (8.62)	**0.034**
Hospital category									
General hospital	876 (97.88)	221 (25.23)	0.286	130 (14.84)	0.510	36 (4.11)	**0.046**	28 (3.20)	0.469
Specialized hospital	19 (2.12)	7 (36.84)	0.286	4 (21.05)	0.510	3 (15.79)	**0.046**	1 (5.26)	0.469
Hospital ownership									
Governmental hospital	882 (98.55)	224 (25.40)	0.748	134 (15.19)	0.235	39 (4.42)	1.000	29 (3.29)	1.000
Private hospital	13 (1.45)	4 (30.77)	0.748	0 (0.00)	0.235	0 (0.00)	1.000	0 (0.00)	1.000
Recent event with significant emotional impact									
No	797 (89.05)	191 (23.96)	**< 0.001**	113 (14.18)	**0.042**	32 (4.02)	0.171	23 (2.89)	0.130
Yes, negative impact	47 (5.25)	23 (48.94)	**< 0.001**	13 (27.66)	**0.042**	4 (8.51)	0.171	3 (6.38)	0.130
Yes, positive impact	51 (5.70)	14 (27.45)	**< 0.001**	8 (15.69)	**0.042**	3 (5.88)	0.171	3 (5.88)	0.130

*Note:* Data are presented as *n* (%). For this analysis, each type of workplace violence was dichotomized as not experienced and experienced at least once. Percentages for workplace violence are row percentages within each category. *p* values were derived from Pearson chi‐square tests or Fisher’s exact tests, as appropriate. Bold *p* values indicate *p* < 0.05.

### 4.5. Correlation Analysis Between Surgical Nurses’ Perception of Holistic Humanistic Caring and Experience of WPV

The correlation between the incidence of various types of WPV experienced by the 895 surgical nurses surveyed and their scores across dimensions of perceived holistic humanistic caring is shown in Table 6. Among these, verbal abuse experienced by surgical nurses was negatively correlated with their perception of patients’ caring, exhibiting the strongest negative correlation.

**TABLE 6:** Correlation analysis results (*r*).

WPV		Self‐caring	Hospital managers’ caring	Department coworkers’ caring	Patients’ caring	Family caring	Friends’ caring	Total score
Verbal violence	Verbal abuse	−0.160^∗∗^	−0.234^∗∗^	−0.232^∗∗^	−0.255^∗∗^	−0.160^∗∗^	−0.163^∗∗^	−0.252^∗∗^
	Verbal threats	−0.140^∗∗^	−0.188^∗∗^	−0.195^∗∗^	−0.187^∗∗^	−0.112^∗∗^	−0.151^∗∗^	−0.206^∗∗^

Physical assault		−0.047	−0.121^∗∗^	−0.145^∗∗^	−0.086^∗^	−0.100^∗∗^	−0.112^∗∗^	−0.129^∗∗^

Sexual harassment		−0.037	−0.100^∗∗^	−0.142^∗∗^	−0.080^∗^	−0.131^∗∗^	−0.118^∗∗^	−0.121^∗∗^

^∗^ *p* < 0.05.

^∗∗^ *p* < 0.01.

## 5. Discussion

In this study, 895 surgical nurses were surveyed, and their scores exceeded 80% on five dimensions of perceived holistic humanistic caring, with only one dimension scoring below this threshold. Among these dimensions, respondents reported the highest level of perceived holistic caring from family, particularly concerning attention to their physical condition. This finding is likely due to the family being a primary environment where nurses engage outside of work, offering substantial opportunities for rest, relaxation, and recovery from physical and mental exhaustion. Conversely, nurses reported the lowest level of perceived caring from the hospital managers, with the lowest‐scoring item being “the hospital has mechanisms for nurse participation in decision‐making.” This indicates a significant deficiency in nurses’ involvement in hospital management, particularly regarding participation channels, and suggests notable shortcomings in the implementation of “shared governance” at the hospitals where the surveyed nurses are employed. Similar findings are reported in studies by Esra Ugur et al., which reveal that hospital management decisions are predominantly influenced by administrative staff and managers, with minimal nurse involvement [37]. Shared governance is a management framework that empowers nurses to participate in decision‐making processes concerning their professional practice environment and the resources that support it [38, 39]. Research indicates that the implementation of shared governance facilitates higher levels of decisional involvement and fosters a sense of empowerment and autonomy among nurses [40, 41]. This approach not only significantly enhances job satisfaction and reduces staff turnover among nurses [42] but also improves patient satisfaction [39]. Given their critical role in the hospital workforce, it is essential for nurses to actively contribute to the institution’s pursuit of high‐quality development. To this end, hospital administrators could encourage nurses to engage in developmental decision‐making processes and establish efficient channels for nurses to express their opinions, such as through the development and enhancement of suggestion or feedback systems. Simultaneously, nursing administrators could evaluate strategies to increase nurses’ involvement in hospital management by identifying factors that facilitate or impede nursing participation in decision‐making and implementing necessary improvements. This entails actively promoting nurses’ engagement in management decisions within the hospital context [43] and providing them with increased decision‐making authority [44].

The results of this study demonstrate that surgical nurses from minority ethnic groups scored significantly lower than their Han ethnic group counterparts across all dimensions of holistic humanistic caring (*p* < 0.05). This disparity may be attributed to the smaller representation of minority ethnic group surgical nurses and the greater geographic distance between their work locations and their hometowns. These findings suggest that hospitals and departments should enhance institutional and departmental support for minority ethnic group surgical nurses to compensate for the lack of familial support at these levels. Similarly, international studies have reported analogous findings: While discrimination against white nurses by patients is rare, minority nurses frequently encounter sexual harassment, racial slurs, disrespect, refusal, or questioning from patients due to factors such as skin color, language barriers, or accents, in contrast to their white counterparts [45, 46]. Consequently, it is imperative for nursing administrators to comprehend the specific circumstances faced by nurses within their departments. For nurses belonging to diverse racial and ethnic backgrounds, or those who are employed at a considerable distance from their hometowns, it is essential that departments offer appropriate support and resources, commensurate with their available capacities.

This study revealed that the overall perception of humanistic caring among surgical nurses in tertiary hospitals was significantly higher than that in secondary hospitals (*p* < 0.05). This disparity may be attributed to the more robust management systems and resource allocation present in tertiary hospitals, which provide an enhanced working environment and greater support for nurses. In China, tertiary hospitals represent the majority of pilot units designated by national and local authorities for the implementation of modern hospital management systems [47]. Compared with secondary hospitals, tertiary hospitals may be better positioned to implement scientifically grounded and innovative staff management models or policies because they generally have stronger organizational structures, performance management systems, and staff development mechanisms [48, 49]. For example, magnet‐related management models emphasize transformational leadership, structural empowerment, exemplary professional practice, innovation, empirical outcomes, and professional autonomy, which are associated with more favorable nursing practice environments, higher job satisfaction, and stronger professional autonomy among nurses [50, 51]. These organizational and managerial advantages may partly explain why nurses in tertiary hospitals reported higher perceptions of holistic humanistic caring in this study.

Regarding departmental factors, thoracic surgery nurses reported lower levels of perceived self‐caring, hospital managers’ caring, family caring, and friends’ caring (*p* < 0.05). This phenomenon may be related to the complex and time‐sensitive nature of thoracic surgical nursing. Patients undergoing thoracic surgery often require intensive perioperative management, including postoperative pain control, respiratory function support, chest tube management, early mobilization, and prevention of pulmonary or cardiac complications [52, 53]. These complex care demands may increase nurses’ workload, perceived urgency, and work pressure. Under sustained workload pressure, nurses may have less time and psychological energy for self‐caring and may be less able to perceive caring from hospital managers, family members, or friends. Therefore, when managing nursing staff in high‐intensity departments such as thoracic surgery, hospital and nursing administrators should strengthen nursing human resource allocation, pay timely attention to nurses’ caring needs, and provide targeted organizational and psychological support. These measures may help alleviate nurses’ stress and promote an overall improvement in their perception of holistic humanistic caring.

In this study, a negative correlation was observed between the overall perceived level of humanistic caring among 895 surgical nurses and their exposure to various forms of WPV (*p* < 0.01). This finding suggests that nurses who have not encountered WPV are more likely to fully perceive the caring provided by their environment compared to those who have experienced such violence. These findings suggest that lower WPV exposure was associated with higher holistic humanistic caring perception among surgical nurses. Reducing WPV exposure may help support nurses’ perception of holistic humanistic caring; however, the direction of this relationship should be further examined in longitudinal studies. The incidence of WPV is an important indicator for evaluating the safety of nursing practice environments, because workplace violence is closely linked to workforce safety, workplace safety culture, burnout, and patient safety culture [1, 54, 55]. Despite this, the prevalence of WPV among nurses remains alarmingly high on a global scale. In China, the incidence rate is reported at 62.4% [56], while in the United States, it is 43% [57]. Furthermore, a study involving 1089 nurses across five European countries found an overall incidence rate of WPV as high as 54% [58].

Consequently, beyond relying on existing national‐level policy safeguards [31, 59], hospitals should prioritize addressing incidents of WPV experienced by nurses [1, 7]. This approach should include the establishment of hospital WPV prevention teams, the provision of educational lectures on prevention and response for newly admitted nurses, the organization of regular safety training sessions, the creation of a safe working environment, and the integration of WPV prevention and response strategies into the daily management of nursing activities [60, 61]. Evidence suggests that education and training interventions can improve nurses’ coping resources and may help reduce the incidence and severity of WPV [61]. Through these measures, hospitals may reduce nurses’ risk of WPV exposure and improve the safety of the nursing practice environment [1, 54, 60, 61]. A safer and more supportive work environment may further enhance nurses’ perceived organizational support and holistic humanistic caring perception, thereby enriching their caring resource system [13, 14]. As a result, they are better equipped to provide humanistic caring to patients, reducing their intention to leave and increasing their job satisfaction. Additionally, research conducted in China suggests that interventions by hospital humanistic caring teams can significantly improve the mental health of nurses who experience WPV [62]. Nurses who have experienced WPV may report lower holistic humanistic caring perception and may require more comprehensive organizational and peer support. Consequently, when delivering caring to these nurses, hospital and departmental managers should intensify efforts to provide organizational and peer support, thereby facilitating psychological recovery and improving their overall health status.

In summary, this study provides preliminary empirical support for the theoretical inference that nurses who perceive more holistic humanistic caring resources may experience a lower frequency of WPV. Humanistic caring is widely recognized as an essential component of nursing practice and patient care [8, 10, 63]. Nonetheless, it is imperative to recognize that humanistic caring, as an individual resource, necessitates ongoing replenishment to maintain its beneficial effects [13, 24]. Such replenishment is instrumental in mitigating the growing strain in nurse–patient relationships, preventing conflicts, and reducing instances of WPV. As a result, it is essential to provide nurses with comprehensive caring from all angles. Strengthening the holistic humanistic caring system for nurses encompasses emotional support from family members and friends, as well as respect and understanding from patients. Hospitals and departments should also strengthen their caring in areas such as psychological support, work assistance, and fostering a collaborative team environment, continuously improving a comprehensive, multi‐tiered system of humanistic caring.

## 6. Strengths and Limitations

This study has several strengths. First, the study included a relatively large sample of 895 valid questionnaire responses, which provided sufficient statistical power for the main analyses and enhanced the reliability of the findings. Second, holistic humanistic caring perception was assessed from multiple dimensions, including self‐caring, hospital managers’ caring, department coworkers’ caring, patients’ caring, family caring, and friends’ caring. This multidimensional assessment allowed a more comprehensive understanding of the caring resources perceived by surgical nurses from both personal and external sources. Third, this study examined different forms of WPV, including verbal abuse, verbal threats, physical assault, and sexual harassment, which provided a more detailed description of surgical nurses’ exposure to WPV. In addition, the study further explored the association between surgical nurses’ WPV experiences and holistic humanistic caring perception, offering empirical evidence for understanding WPV from the perspective of caring resources.

Several limitations should also be acknowledged. First, the cross‐sectional design limits the ability to infer causal relationships between holistic humanistic caring perception and WPV. Therefore, the negative associations observed in this study should be interpreted as correlational rather than causal. Second, although the sample size was relatively large, participants were recruited using a non‐probability sampling method, which may limit the generalizability of the findings to all surgical nurses in China. Third, some subgroups were relatively small, such as nurses from certain departments, private hospitals, and those with senior professional titles, which may have affected the stability of subgroup comparisons. Future studies should adopt longitudinal or multicentre designs with probability sampling where possible, combine self‐reported data with institutional reporting records, and further explore the mechanisms linking humanistic caring resources with WPV.

## 7. Implications for Nursing Management

Hospital administrators should give full attention to nurses’ caring needs, translating concern for nurses into concrete policies and daily practices. Nurses should be encouraged to contribute their insights to hospital management, offering personal expertise and wisdom for institutional development. Regular channels should be established for nurses to provide suggestions and recommendations. Concurrently, nursing administrators should prioritize nurses’ humanistic caring needs by proactively establishing multidimensional support systems to elevate their holistic humanistic caring perception. This can be achieved through fostering a collaborative team atmosphere, conducting regular mental health support activities, optimizing shift schedules, and promoting positive communication and cooperation among colleagues to enhance nurses’ sense of belonging and fulfillment. Efforts should be made to actively promote understanding and respect for the nursing profession among patients and the general public, fostering harmonious nurse–patient relationships. This helps mitigate the risk of adverse events experienced by nurses in their work. The goal is to reduce WPV while simultaneously increasing nurses’ job satisfaction and professional resilience, thereby providing a strong foundation for delivering high‐quality nursing caring and enhancing patient satisfaction.

## 8. Conclusion

To our knowledge, this study is among the first to examine the association between nurses’ holistic humanistic caring perception and their experience of WPV. In China, surgical nurses’ overall perception of humanistic caring is at a moderately high level, with the highest perception regarding family caring and the lowest regarding hospital managers’ caring—particularly in terms of participation in hospital management. This suggests hospitals should further streamline channels for nurse participation in management, genuinely implement “shared governance,” and encourage nurses to contribute suggestions for hospital development. This approach would enhance job satisfaction, strengthen a sense of belonging, and reduce turnover rates. Additionally, nurses’ holistic humanistic caring perception was significantly negatively associated with their experience of all types of WPV. This finding suggests that nurses who perceived more humanistic caring from various sources tended to report a lower frequency of WPV experiences. These results provide preliminary empirical support for the theoretical inference that holistic humanistic caring perception and WPV experiences are related. However, because this study used a cross‐sectional design, this association should be interpreted as correlational rather than causal. The findings further suggest that nurses who have experienced WPV should receive more comprehensive humanistic caring to alleviate psychological trauma and promote health recovery. Future longitudinal or intervention studies are needed to clarify whether enhancing nurses’ holistic humanistic caring resources can reduce WPV exposure and improve nurses’ caring capacity.

## Author Contributions

Hongwei Chang, Danli Xiong: Conceptualization, formal analysis, and writing ‐ original draft; Min Tian, Ting Chen, Yao Cheng, Yan Meng, Huantian Li: Data collection; Yuqin Chen: The primary designer of the questionnaire; Sai Yang, Yilan Liu: Validation and writing ‐ review and editing.

## Funding

This study was supported by the Natural Science Foundation of Hubei Province, China, under Grant No. 2023AFB910. The funder had no role in the study design, data collection and analysis, decision to publish, or preparation of the manuscript.

## Conflicts of Interest

The authors declare that there are no conflicts of interest regarding the publication of this paper.

## Data Availability

Data are available upon request due to privacy/ethical restrictions.
